# Intermittent Fasting: From Calories to Time Restriction

**DOI:** 10.1093/geroni/igab046.440

**Published:** 2021-12-17

**Authors:** Rafael de Cabo

**Affiliations:** NIA, Baltimore, Maryland, United States

## Abstract

Classic implementation of calorie restriction (CR) in laboratory animals increases health and longevity in most model organisms. Traditionally, chronic CR is the reduction of daily energy intake without malnutrition. Recently, paradigms have emerged that recapitulate some of the beneficial aspects of this intervention, avoiding some of its challenges. The length of daily fasting length and periodicity have emerged as potential drivers behind CR’s beneficial health effects. Numerous strategies and eating patterns, including prolonged periods of fasting, have been successfully developed to mimic many of CR’s benefits without its austerity. These new feeding protocols range from short mealtimes designed to interact with our circadian system (daily time-restricted feeding) to more extended fasting regimens known as intermittent fasting. We will discuss the current status of knowledge on different strategies to reap the benefits of CR on metabolic health in rodent models and humans without the rigor of chronic reductions in caloric intake.

